# Desmoplakin Cardiomyopathy: Myocarditis-Like Episodes

**DOI:** 10.7759/cureus.87311

**Published:** 2025-07-05

**Authors:** Tarek Fatrous, Sara Ibzea, Sharafath Hussain Zahir Hussain

**Affiliations:** 1 Medicine, Milton Keynes University Hospital NHS Trust, Milton Keynes, GBR; 2 Medicine and Surgery, Milton Keynes University Hospital NHS Trust, Milton Keynes, GBR

**Keywords:** cardiomyopathy, covid, desmoplakin, genetics, myocarditis, vaccine

## Abstract

We report a case of a 21-year-old woman who presented with recurrent chest pain over a three-year period, initially attributed to myocarditis. She first presented with elevated troponin levels and myocardial oedema on cardiac magnetic resonance imaging (MRI), consistent with acute myocarditis. Despite symptomatic management and a gradual return to activity, she experienced multiple relapses characterised by chest pain, subtle left ventricular dysfunction, and persistent myocardial scarring on serial MRI, but without active inflammation. A multidisciplinary evaluation and genetic testing revealed a heterozygous deletion in the desmoplakin (DSP) gene, confirming the diagnosis of desmoplakin cardiomyopathy. Electrocardiographic abnormalities included T-wave inversions in the anterior and lateral leads. Due to her elevated risk of sudden cardiac death, she underwent implantation of a subcutaneous implantable cardioverter-defibrillator (s-ICD) for primary prevention. This case highlights that DSP cardiomyopathy should be considered in the differential diagnosis of recurrent myocarditis-like presentations, particularly in young individuals with non-ischaemic findings and suggestive imaging features.

## Introduction

Myocarditis is a prevalent cause of acute chest pain and is routinely included in the differential diagnosis for such presentations. It is characterized by inflammatory injury to the myocardium, most often due to viral infections, though it can also arise secondary to autoimmune diseases like systemic lupus erythematosus. Management is typically conservative, focused on supportive care and analgesics, with most patients achieving full recovery [1].

In rarer instances, arrhythmogenic cardiomyopathy (ACM) can present with myocarditis-like chest pain, a phenomenon referred to as the “hot phase.” The latter is defined as a phase of inflammation and chest pain resembling acute myocarditis in genetic cardiomyopathies.

This presentation is an important diagnostic consideration alongside acute coronary syndromes and viral myocarditis [2]. Genetic predispositions play a major role in ACM: Bariani et al. reported that 77% of ACM cases have an identifiable genetic mutation, with the desmoplakin (DSP) gene being the most frequently implicated [2]. The same study reported an incidence of DSP cardiomyopathy of 14-22%.

Here, we report the case of a young adult female who initially presented with acute myocarditis at age 17 and was ultimately diagnosed with DSP cardiomyopathy in 2024 after multiple recurrent episodes.

## Case presentation

A 21-year-old female with no prior medical history or significant family history presented with recurrent episodes of chest pain over a three-year period. Her first presentation was on September 3, 2021 (age 17), with a 24-hour history of stabbing central chest pain. Notably, she had received a Pfizer COVID-19 vaccine on August 21, 2021, and had experienced intermittent chest pain and fatigue since that time. It is important to note that although the initial presentation occurred shortly after COVID-19 vaccination, subsequent evaluation revealed a genetic etiology unrelated to the vaccine.

On admission, laboratory tests showed a markedly elevated troponin-T level of 17,558.3 ng/L (reference range 2.3-11.7 ng/L) and a mild leukocytosis of 12.4×10^9/L (reference range 3.7-11.1×10^9/L). Her electrocardiogram (ECG) on presentation was unremarkable. Repeat troponin levels on September 4 and 5 demonstrated a downtrend (12,725.7 ng/L and 1,917.3 ng/L, respectively). A working diagnosis of post-vaccine acute myocarditis was made. She was treated with ibuprofen 400 mg three times daily and colchicine 500 μg twice daily. A transthoracic echocardiogram was unremarkable. The patient’s chest pain improved, and she was discharged on September 7, 2021, with a six-week course of colchicine and ibuprofen (with lansoprazole for gastroprotection), activity modification (avoidance of intense exercise), and outpatient follow-up including a planned cardiac magnetic resonance imaging (MRI) scan.

**Figure 1:**
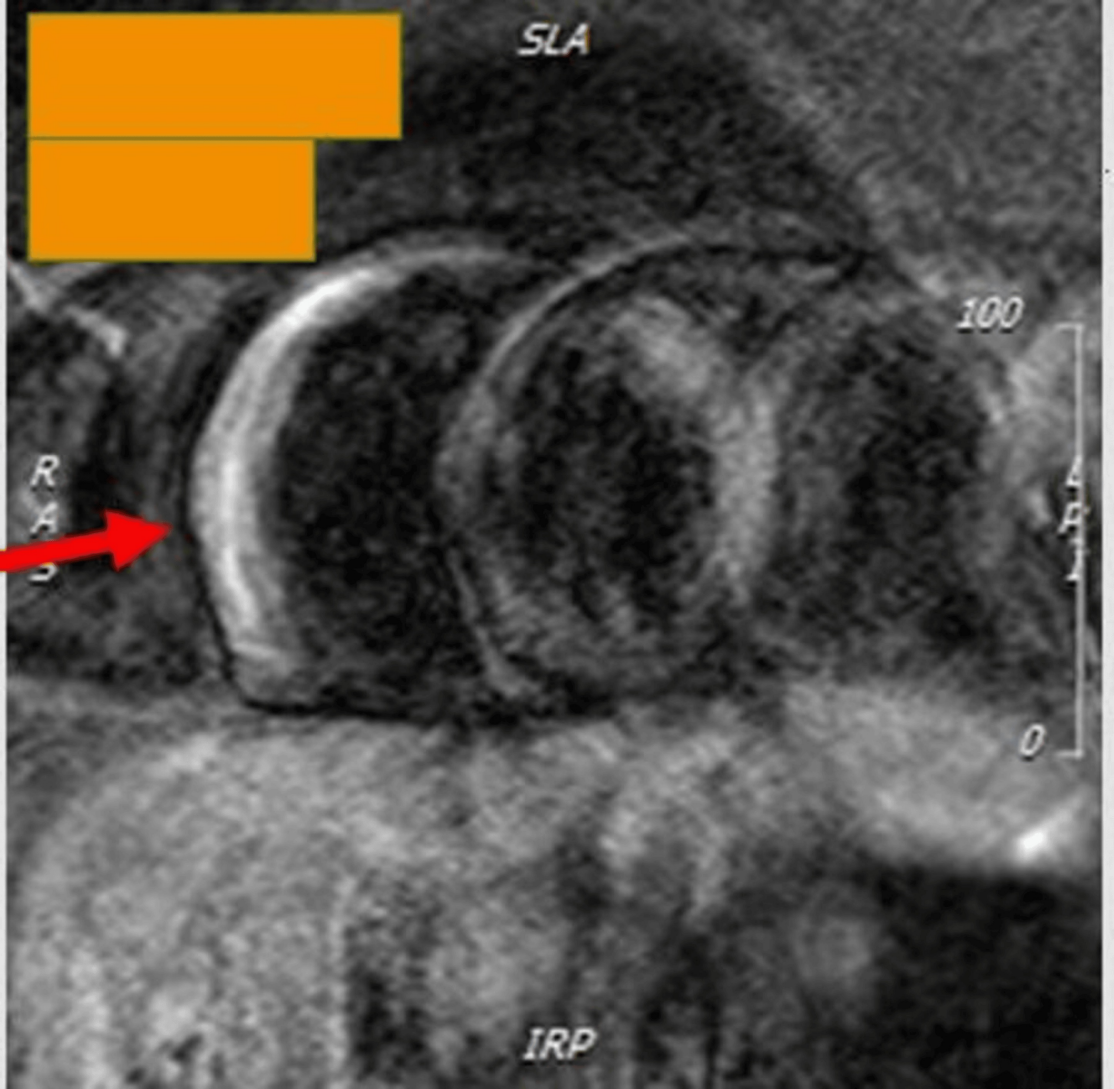
Cardiac MRI (2021). Note the myocardial edema.

Cardiac MRI performed on September 24, 2021 (Figure 1) confirmed recent myocarditis, demonstrating myocardial edema and extensive subepicardial late gadolinium enhancement in the septal, inferior, and inferolateral walls. Left ventricular (LV) volumes were low-normal, with mildly reduced systolic function (LV ejection fraction (LVEF) 56%). Her medical regimen was maintained, and a gradual return to activity was advised.

The patient had a second presentation on January 9, 2022, with recurrent chest pain. This time, troponin-T was within normal limits (4.4 ng/L) and all other labs and ECG were unremarkable. She described intermittent chest discomfort with a two-day continuous episode prior to presentation. Given the normal investigations and improving symptoms, she was reassured and discharged with advice to return if symptoms worsened.

During a routine cardiology clinic review on September 6, 2022 (age 18), the patient reported ongoing episodic chest pain and expressed psychological distress about her inability to participate in sports. Ramipril (1.25 mg daily) and bisoprolol (1.25 mg daily) were initiated for cardioprotective therapy, and a repeat cardiac MRI was scheduled.

**Figure 2:**
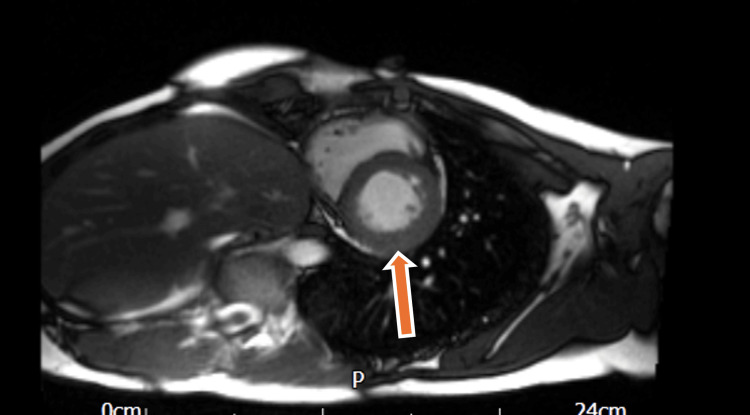
Cardiac MRI (2022)

The follow-up MRI on November 15, 2022 (Figure 2) showed no new myocardial edema but persistent, patchy subepicardial scar in the inferior LV wall and the right ventricular aspect of the septum and inferolateral wall - consistent with prior myocarditic injury. LV systolic function remained low-normal (LVEF 56%) with no evidence of active inflammation.

**Figure 3:**
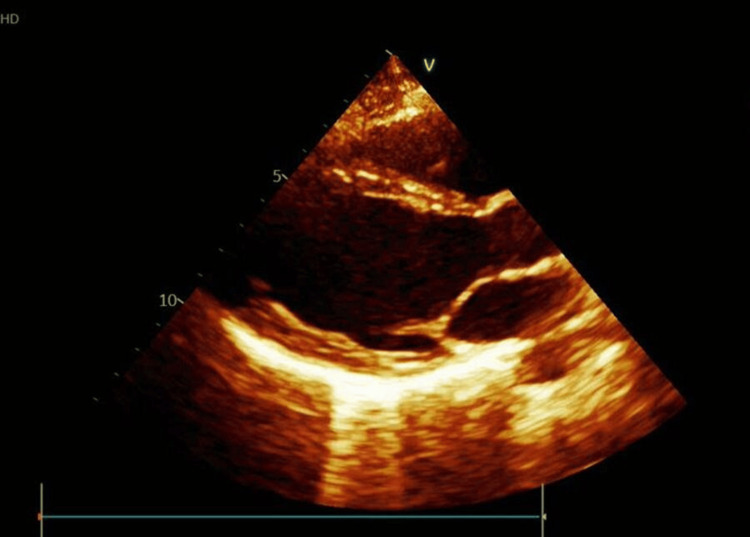
Echocardiogram.

At 19 years of age (May 9, 2023), the patient continued to experience similar chest pain episodes accompanied by fatigue and exertional dyspnea. Ambulatory Holter monitoring and echocardiography (Figure 3) were conducted, which revealed no arrhythmias or structural abnormalities; formal cardiac rehabilitation was deemed unnecessary.

In September 2023, now age 20, she presented again with a 72-hour history of persistent chest pain. Troponin-T was mildly elevated at 898 ng/L. She was admitted for observation and treated with colchicine and ibuprofen. A repeat echocardiogram during this admission remained normal. Troponin levels trended down to 47.7 ng/L by the second hospital day and 13.3 ng/L two days later. An extensive autoimmune workup (including antinuclear antibody (ANA), rheumatoid factor, and inflammatory markers) was unremarkable. Given her recurrent episodes, a third cardiac MRI was obtained.

**Figure 4:**
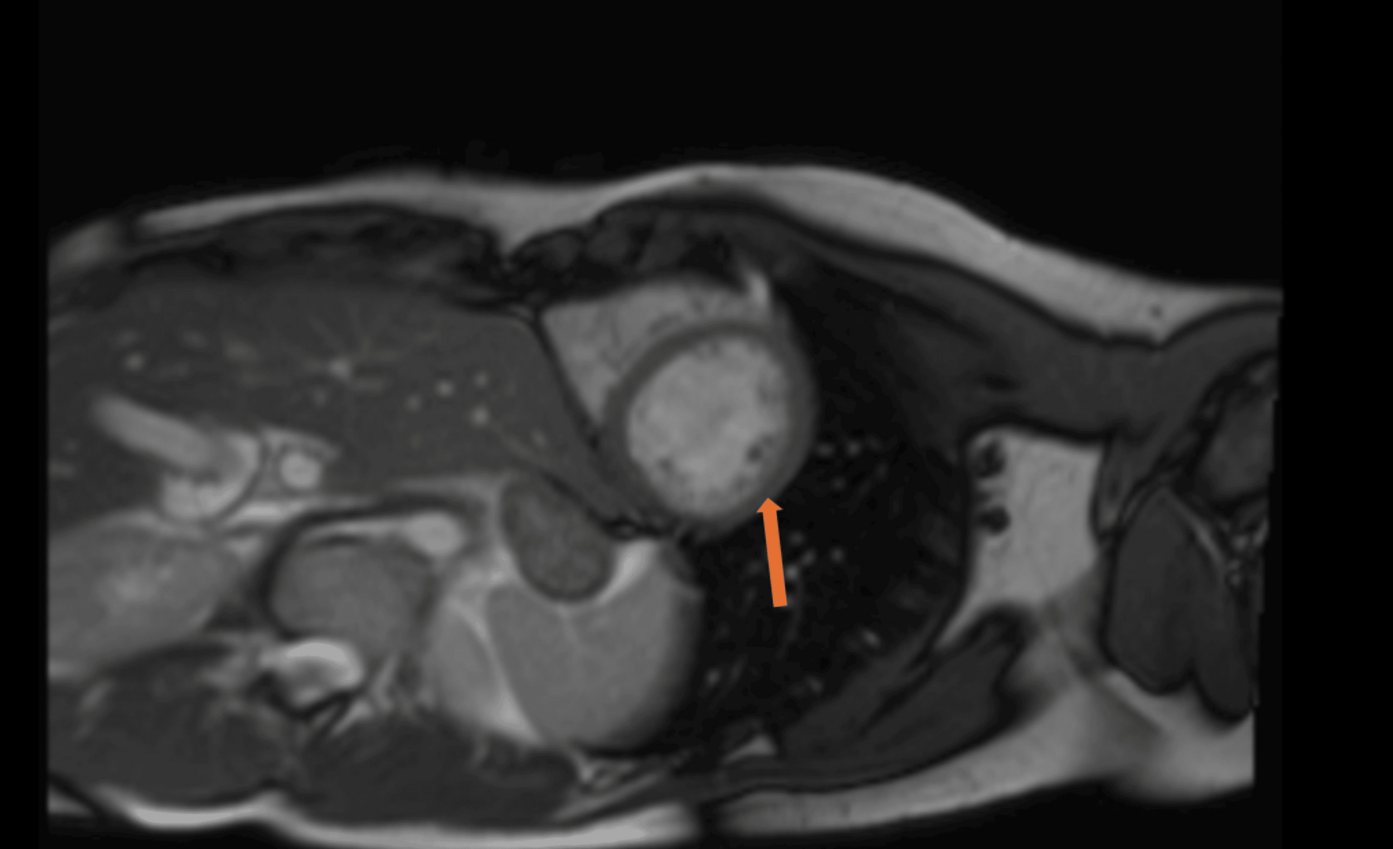
Cardiac MRI (2023)

The MRI on September 17, 2023 (Figure 4) revealed slightly worsening LV systolic impairment (LVEF 51%) which remained within low-normal range. There were no signs of active myocarditis, but the pattern of subepicardial scar seen on previous imaging persisted.

In light of her recurrent myocarditis-like episodes and residual myocardial scarring, a multidisciplinary team (cardiology, genetics, and electrophysiology) discussion was held on September 20, 2023. Genetic testing, done via a 43-gene inherited cardiomyopathy panel, was recommended and performed, which identified a heterozygous nonsense variant in the DSP gene: c.3805C>T p.(Arg1269Ter) which is predicted to cause premature protein truncation leading to loss of function - an established disease mechanism of this gene. This confirmed the diagnosis of desmoplakin cardiomyopathy and indicated a heightened risk for arrhythmias and sudden cardiac death. In February 2024, the patient underwent successful implantation of a subcutaneous implantable cardioverter-defibrillator (s-ICD) for primary prevention of lethal arrhythmias. The timeline of events is shown in Table 1.

**Table 1:** Timeline of Events LVEF: left ventricular ejection fraction, DSP: desmoplakin, ICD: implantable cardioverter-defibrillator, MDT: multidisciplinary team

Date	Events
September 2021, First Presentation	Central chest pain and had Pfizer vaccine two weeks prior. Elevated troponins but normal ECG, treated as myocarditis and discharged with colchicine and ibuprofen. MRI confirmed evidence of recent myocarditis.
January 2022, Second Presentation	Chest pain with normal ECG and troponin levels. Reassured and discharged.
September 2022, Cardiology Outpatient	Started on ramipril and bisoprolol for cardioprotection.
November 2022, Second MRI	Similar appearances to first MRI in 2021.
September 2023, Third presentation	72 hours of persistent chest pain with elevated troponin levels. Decision made for autoimmune work-up and third MRI which showed an LVEF 51%.
September 2023, MDT discussion	Genetic testing completed which identified the mutated DSP gene.
February 2024	ICD insertion.

The patient continues to be followed closely by cardiology. She remains on ramipril and bisoprolol and has had no further chest pain episodes since the s-ICD placement. It is also notable that her 24-year-old brother underwent genetic screening in early 2024 due to his diagnosis of postural orthostatic tachycardia syndrome (POTS); he was found to carry the same DSP mutation, prompting cardiac evaluation and surveillance in that family member.

## Discussion

This case highlights an infrequent etiology of recurrent myocarditis, desmoplakin cardiomyopathy (DSP-CM), in a young patient. The association between DSP gene mutations and cardiomyopathy was first described by Rampazzo et al. in 2002, who identified a DSP mutation segregating with arrhythmogenic cardiomyopathy in an Italian family [3]. Since then, multiple studies have established a strong correlation between DSP mutations and adverse cardiac outcomes, including arrhythmia and even presentations that mimic acute myocardial infarction (such as chest pain with ST-elevation despite normal coronary angiography) [4].

There is a well-documented link between DSP mutations and progressive ventricular dysfunction. For instance, our patient’s LV ejection fraction declined from 56% to 51% over two years, reflecting subtle LV impairment consistent with what has been reported in DSP-CM [5]. Many patients with DSP-CM experience an initial inflammatory or myocarditis-like phase of disease (the “hot phase”). Bariani et al. described this phenomenon, noting that approximately 5% of arrhythmogenic cardiomyopathy patients present with myocarditis-like episodes; among those, DSP mutations accounted for 69% of cases [2-6].

Electrocardiographic abnormalities are another hallmark of DSP-CM. Studies frequently report T-wave inversions in the anterior and lateral precordial leads, although the precise distribution can vary. This pattern was evident in our patient, who demonstrated T-wave inversions in leads V_2-V_3 and V_5-V_6 during follow-up evaluations [7].

Management of DSP cardiomyopathy remains challenging, as evidence-based guidelines are limited. A personalized, multidisciplinary approach is advised, tailored to the extent of myocardial fibrosis, arrhythmic burden, and disease progression in each patient. In our patient’s case, the disease was identified at a relatively early stage with mild fibrosis and no sustained arrhythmias; thus, an s-ICD was implanted for primary prevention in line with 2023 European Society of Cardiology recommendations for arrhythmogenic cardiomyopathies. She was also maintained on guideline-directed medical therapy (anangiotensin-converting enzyme inhibitor and beta-blocker). In advanced cases of DSP-CM where there is significant ventricular scarring or ongoing inflammation, immunosuppressive therapy (such as corticosteroids, methotrexate, or mycophenolate) has been reported as a therapeutic consideration [8,9]. However, the definitive treatment strategy for DSP-CM is not established, and further research, including prospective trials, is needed to optimize care for these patients.

Recent literature continues to shed light on this entity. For example, a multicenter study of pediatric DSP cardiomyopathy patients found that severe biventricular disease confers a high risk of malignant arrhythmias, reinforcing the importance of vigilant monitoring and timely ICD placement in high-risk individuals [10]. The overlap between myocarditis and DSP-related cardiomyopathy is further highlighted in the recent pediatric cohort data showing that nearly half of young patients with DSP variants were initially diagnosed with myocarditis before the genetic cause was recognized [10].

Moreover, emerging evidence points to a broader role of genetic predisposition in myocarditis: up to 16% of myocarditis cases may have an underlying pathogenic variant in a cardiomyopathy gene, with DSP mutations being a notable contributor to recurrent “inflammatory” cardiomyopathy [11]. Recognizing such genetic influences is crucial for guiding surveillance and therapy in patients with recurrent myocarditis.

## Conclusions

Desmoplakin cardiomyopathy is an uncommon but important cause of recurrent myocarditis in young patients. Its diagnosis can be difficult due to overlapping features with more common conditions like viral myocarditis and acute coronary syndromes. As illustrated by this case, recognizing the pattern of recurrent myocardial injury and pursuing genetic evaluation can be lifesaving. Ongoing research into the pathophysiology and optimal management of DSP-CM is needed, but clinicians should maintain a high index of suspicion for this condition in patients with unexplained recurrent chest pain or myocarditis-especially if there is a family history of arrhythmogenic or sudden cardiac death. Early identification of DSP-CM allows for appropriate interventions, such as ICD placement and tailored therapy, to prevent adverse outcomes.
